# Implementing simulation in a nursing education programme: a case report from Tanzania

**DOI:** 10.1186/s41077-017-0048-z

**Published:** 2017-10-03

**Authors:** Ingrid Tjoflåt, Bodil Bø Våga, Eldar Søreide

**Affiliations:** 1 Faculty of Health Sciences, University of Stavanger, 4036 Stavanger, Norway; 2 Stavanger University Hospital, Helse Stavanger HF, Postboks 8100, 4068 Stavanger, Norway

**Keywords:** Simulation-based education, Nursing education, Education in a low-income context, Nurse

## Abstract

This paper presents a description of, and some reflections around, the experience of implementing simulation-based education within a nursing education programme in a low-income context. The students in the nursing education programme found the simulation sessions to be useful, motivating and a realistic learning method. Our experience may provide useful insight for other nursing education programmes in low-income contexts. It looks like a deeper knowledge about the feasibility of simulation-based education from both the teacher and student perspective is necessary.

## Introduction

This paper will describe and reflect upon the experiences of implementing simulation-based education in a nursing education programme in a low-income context in Tanzania. This was a cooperative project between nursing teachers at a local nursing school located in a highly rural area in north-central Tanzania and nursing teachers from a Norwegian nursing school. These two nursing schools have a history of long-term collaboration through a student and staff exchange programme.

The nursing school in Tanzania enrols approximately 100 nursing students every year, with a total of 223 nursing students and 24 administrative and academic staff members. The school is regulated by the national curriculum for the Ordinary Diploma Programme in Nursing developed by the Ministry of Health and Social Welfare in Tanzania. Simulation-based education is defined as one of the teaching methods within the national curriculum. Although the school has a new skills training and simulation centre and the teachers have knowledge about the simulation method, simulation-based education has not yet been implemented. However, the Norwegian nursing school has used simulation-based education for several years in their Bachelor of Nursing programme. Supporting the nursing school in Tanzania to implement simulation-based education was mutually agreed upon as a collaborative project, and the two scenarios for the simulation were jointly selected.

## Background

Simulation-based education, aiming to bridge the gap between theory and practice through innovative teaching strategies, is described as a key component in nursing students’ learning and preparation for practice and professional life [1–3]. This pedagogical method has been documented to be useful, beneficial and effective for students in their learning processes, and the results have been addressed as mainly positive [4–6]. Research has pointed out how the students’ knowledge and confidence increased after simulation-based education. Moreover, students have expressed satisfaction with simulation as a pedagogical method when compared to other teaching and learning strategies [2, 6].

As a pedagogical method, simulation enables students to learn how to reconcile theory with practice through role-playing and case studies. The students work together in a supportive skills training environment [2], while the teachers/staff facilitate the simulation sessions. Before role-playing, the facilitator introduces the setting and the case, preparing the students and answering any questions, and then maintains a withdrawn role during the role-playing/case study. After the simulation, a debriefing session is an important phase of learning for the students. Promoting reflection during the debriefing is important for learning in simulation-based education [7].

While simulation-based education has increased in both extent and scope in many nursing education programmes in Europe, the USA, Asia, the Middle East and Australia, the literature shows a lack of implementation and research on simulation in low- and middle-income countries [2, 8–14]. To the best of our knowledge, no studies have highlighted simulation-based education in nursing education programmes in Sub-Saharan Africa.

## The simulation experience

The simulation sessions were conducted with 27 third-year nursing students; three of them were Norwegian students participating in a student exchange programme. Three scenario sessions, with eight to nine students in each session, were carried out in 1 day. The simulation sessions took place in the skills training and simulation centre in the nursing school. Two different scenarios were selected: (1) a patient case concerning postoperative care and (2) a patient case concerning sepsis in the emergency room.

Overall, the simulation method was new to the Tanzanian students and teachers; therefore, the Tanzanian teachers observed, while the Norwegian teachers facilitated the simulation sessions. The Norwegian nursing students had participated in simulation-based education sessions in Norway; however, this was their first simulation session together with the Tanzanian nursing students. To reduce anxiety and create a relaxed atmosphere, each session began with the facilitator briefly explaining that the simulation sessions were not graded and that the experience was about learning. The learning outcomes in both scenarios included the students conducting a primary survey of the patient, implementing relevant nursing interventions and working as a team. Descriptions of the patient’s case, learning outcomes and basic nursing equipment, such as blood pressure (BP) cuffs, intravenous (IV) lines and stethoscopes, were presented to the students during the briefing phase. In each case, one student played the role of the patient and was instructed about how to act in the scenario. Three students participated in the scenario as nurses, while the rest of the group observed the simulation session. In the sepsis scenario, one student also acted as a relative of the patient. The observers were all presented with specific objectives to look for. One group observed how the primary survey was conducted, and the second group looked at the communication and collaboration during the simulation. Each scenario session lasted approximately 10–12 min, followed by a debriefing session lasting for about 30 min.

The debriefing phase started with the students describing what happened during the scenario. This was followed up with the analytical phase, in which the students were asked what was successful in the scenario and why, and what could have been improved. In the last phase of the debriefing session, the students were asked to express what they had learned during the simulation session and how they could transfer this knowledge into clinical practice. We carried out the debriefing phase based on our long existing “good practice” in simulation-based education. Multiple theoretical debriefing methods exist [7], and when we retrospectively reflected on our debriefing methodology, we learned that our practice was aligned with Arora et al.’s objective structured assessment of debriefing [15].

To strengthen the learning outcomes, the same scenario was run twice, with those students who actively participated in the scenario the first time, playing the role of observers the second time. After the simulation session, the students were asked to complete a faculty-derived evaluation form describing what they learned in the simulation session, whether the simulation helped them acquire skills useful for clinical practice, what they liked about participating in the simulation sessions and what they thought of the simulation scenarios.

## Students’ simulation experiences

The students’ comments on the evaluation forms were overwhelmingly positive. They reported that they had learned (1) the importance of teamwork and communication; (2) the principles of airway, breathing and circulation; and (3) to take a full patient history before contacting the doctor. Some of them also mentioned (4) the important role that nurses played in assessing a patient. Some of the other learning points described were as follows: significance of hand hygiene, availability of proper equipment, documenting the nursing care and caring for relatives in an acute situation. These experiences were consistent with the descriptions of previous studies [4–6]. In a setting where nurses are known for and referred to as being task-oriented [16, 17], it was interesting to see that the local nursing students emphasized communication and teamwork as particularly important areas of learning through the simulation-based learning.

The students reported that the simulation was “good” and that they wanted more opportunities for simulation-based education. They explained that they found the simulation session to be a “real situation” and that it showed them how to carry out proper care. One student said that the “Simulation increase out capacity to learn, because it is done by acting out real situations in caring for the patients”. Moreover, they explained that in the simulation sessions, they were able to act as nurses, including communication with the team, doctor and relative to provide good care. The nursing students also stated that the simulation sessions helped them to understand the nursing care and interventions to be provided, prioritizing care, relating theory to practice and improving their knowledge and skills in a specific case. These experiences were in accordance with the previous research, which reported that simulation-based education is a key component in a nursing student’s preparation for practice and a professional life [1–3].

Several students stated that the debriefing sessions were particularly helpful. The debriefing session after simulated role-playing is an important phase of student learning [7]. According to one student, “The evaluation session after the simulation was where I got good input on my improvement”. Another student said, “I liked the evaluation and supervision because it helped me to understand the things that were new to me and how they should be taken care of in real situations”. Through the debriefing sessions, the students were able to understand that simulation-based education is beneficial and may improve clinical practice. The feedback highlighted how the nursing students experienced the simulated cases as “real settings”.

## Further implications

The experience of implementing simulated-based education within a nursing education programme in a low-income context in Tanzania was very positive and was reported to be beneficial for the nursing students. The experiences described may provide insights for other nursing education programmes in low-income contexts, although additional research is necessary. The local nursing education teachers were concerned with the issue of personnel resources, since there were very few teachers when compared to the number of nursing students. However, they reflected on the fact that for this project, we were able to carry out simulations with 27 students in 1 day, with the presence of only one teacher. Discussions with the nursing teachers revealed that they were very positive about continuing to develop and implement simulation-based education. Further exploration of the possibility of replacing classroom teaching with simulation-based education will provide important knowledge about nursing student education programmes in settings where resources are scarce, in terms of both personnel and professional equipment.

## Conclusion

This paper has attempted to make sound reasons for the present implementation of simulation-based nursing education. It can also guide and encourage the further development of new pedagogical methods in settings where resources are scarce in terms of both staff and professional equipment.
